# Association between metabolic syndrome and mortality among breast cancer survivors: a prospective cohort study

**DOI:** 10.3389/fendo.2026.1854729

**Published:** 2026-08-03

**Authors:** Ting Zhang, Xiaoni Cai, Shaoqing Qian, Jingxuan Huang

**Affiliations:** 1 Department of Breast Surgery, Shaoxing Shangyu People’s Hospital, Shaoxing, Zhejiang, China; 2 Department of Plastic and Reconstructive Surgery, Shanghai Ninth People’s Hospital, Shanghai Jiao Tong University School of Medicine, Shanghai, China

**Keywords:** breast cancer, breast cancer survivor, cancer survivor, cancer survivorship, metabolic syndrome

## Abstract

**Background:**

Metabolic syndrome (MetS) has been associated with elevated mortality risk and cardiovascular disease incident among the general population. However, evidence on its impact among breast cancer (BC) survivors remains limited.

**Methods:**

BC survivors in the UK Biobank, a large-scale, population-based prospective cohort, were included in this study. MetS was defined as fulfilling at least three of the following criteria: (1) central obesity, (2) elevated triglycerides or taking lipid-lowering medication, (3) increased blood pressure, (4) elevated fasting blood glucose, and (5) reduced HDL cholesterol. The primary outcome was all-cause mortality, while the secondary outcomes were comorbidity incident. The associations between MetS with mortality and comorbidity incident were determined using Cox proportional regression by quantifying the hazard ratio (HR) and its 95% confidence interval (CI).

**Results:**

After a median follow-up of 15.7 (interquartile range, 15.1 to 16.4) years, a total of 1,251 deaths were observed among 6,027 BC survivors, of whom 1,997 (33.1%) had MetS. MetS was significantly associated with all-cause mortality (HR, 1.23; 95% CI, 1.08 to 1.41, *p* = 0.002). Meanwhile, MetS was also significantly associated with elevated risk of myocardial infarction (MI; HR, 1.56; 95% CI, 1.02 to 2.40), atrial fibrillation (AF; HR, 1.61; 95% CI, 1.04 to 2.49), and chronic kidney disease (CKD; HR, 1.98 95% CI, 1.56 to 2.51) incident. Fatty acid metabolism and inflammation were identified as the potential mediating factors between MetS and mortality.

**Conclusion:**

MetS was significantly associated with elevated mortality risk and incident of MI, AF, and CKD. The MetS–mortality association was potentially mediated by fatty acid metabolism and inflammation. Findings highlighted MetS as a potentially modifiable risk factor in BC survivors, underscoring the need for targeted metabolic management to improve long-term outcomes.

## Introduction

Metabolic syndrome (MetS) is defined as the presence of at least three of the following criteria: elevated waist circumference (i.e., central obesity), triglyceride level, blood glucose, and blood pressure, and reduced HDL cholesterol (1). Approximately 25% of the global population had MetS (2), while 40% of the individuals aged older than 60 years met the diagnosis criteria in the US (3). MetS has been reported to be associated with a diverse adverse health outcome, including increased mortality (4) and elevated risk of cardiovascular disease (5), cancer (6, 7), and kidney disease (8).

Breast cancer (BC) survivors represent a unique and growing population that has transitioned from the acute phase of cancer treatment to long-term survivorship, facing distinct health challenges compared to the general population (9, 10). Moreover, BC treatment frequently involves prolonged treatment, especially adjuvant endocrine therapy (11), which can adversely affect lipid metabolism, insulin sensitivity, and body composition (12). In addition, survivorship is frequently accompanied by reduced physical activity, altered body composition, and chronic inflammation (13, 14). BC survivor’s overall mortality risk is driven not only by cancer recurrence but also by cardiovascular and metabolic complications. Nevertheless, evidence regarding the association between MetS and mortality risk in this specific population remains limited. Clarifying this relationship is essential for informing integrated, personalized survivorship care strategies.

Here, in this study, we aimed to examine the association between MetS and mortality among BC survivors. We also sought to test the association between MetS with a diverse comorbidity incident among these survivors. In addition, we tested the metabolomes associated with MetS and mediating the mortality association. Together, these findings provide integrated epidemiological and metabolic evidence linking MetS to adverse prognosis in BC survivors, highlighting potential mechanistic pathways and targets for risk stratification and intervention.

## Methods

### Study participants

UK Biobank is a population-based, prospective, longitudinal cohort study that recruited more than 500,000 participants from 22 centers across England, Scotland, and Wales during 2006–2010 (15). Touchscreen questionnaires, physical examination and measurement, and electronic records were used to collect participants’ sociodemographic characteristics, lifestyle variables, medical history, cancer diagnosis, and death records. Details of the UK Biobank have been introduced elsewhere (15). BC survivors were defined as those participants who were diagnosed with BC (ICD-10 code C50) beyond 1 year at study recruitment (16). Male survivors and those without complete MetS assessment were excluded (**Supplementary Figure 1**). Data utilized in this study were obtained under application 195606 and approved by the Ethics Committee of Shaoxing Shangyu People’s Hospital (No. 2023126).

### Metabolic syndrome ascertainment

MetS was assessed at study entry and defined as the presence of ≥3 of the following criteria: (1) central obesity (waist circumference ≥88 cm), (2) elevated triglycerides (≥1.7 mmol/L) or taking lipid-lowering medication, (3) increased blood pressure (≥130 mmHg systolic blood pressure or ≥85 mmHg diastolic blood pressure) or taking hypertension medication, (4) elevated fasting blood glucose (≥5.6 mmol/L) or taking blood glucose medication, and (5) reduced HDL-cholesterol (<1.3 mmol/L) (1). BC survivors were then classified into two groups according to with or without MetS.

### Outcomes

The primary outcome of this study was all-cause mortality. Death records were captured from the National Health Service (NHS) England for survivors in England and Wales and the NHS Central Register for survivors from Scotland. Death causes were identified using ICD-10 codes. Survivors were followed since study entry and ended until death or end of follow-up (30 November 2024), whichever came first. The secondary outcomes including stroke, myocardial infarction (MI), atrial fibrillation (AF), heart failure (HF), chronic obstructive pulmonary disease (COPD), chronic kidney disease (CKD), and depression. Disease diagnosis cases were collected by linking with hospital inpatient record, primary care data, and death registry, and were ascertained using ICD-10 code.

### Covariates

Sociodemographic characteristics and lifestyle variables that might impact the association between MetS and outcomes were collected at baseline, including age at study recruitment, ethnicity (White vs. non-White), Townsend Deprivation Index (TDI), education level (college vs. others), assessment center (England vs. Scotland/Wales), body mass index (BMI), smoking status (never vs. pervious or current), alcohol status (current vs. pervious or never), physical activity [low vs. moderate vs. high vs. missing, classified according to International Physical Activity Questionnaire (IPAQ)], prevalent diagnosis (yes vs. no) of cardiovascular disease, COPD, CKD, and cancer survivorship duration.

### Metabolomes

Plasma metabolites were measured using high-throughput nuclear magnetic resonance (NMR) platform (Nightingale Health Plc., Helsinki, Finland). The details have been described elsewhere (17). A total of 249 biomarkers were measured, of which 81 were derived ratios. Survivors with missing biomarker data were excluded in the mediation analysis.

### Statistical analysis

Continuous variables were summarized in median [interquartile range (IQR)] or mean [standard deviation (SD)], as appropriate, while categorical variables were summarized using frequency and percentage. Baseline characteristics were demonstrated stratified by MetS status, and compared using *t*-test, Wilcoxon test, or chi-square test, as appropriate.

The association between MetS with mortality and comorbidities were tested using Cox proportional regression model. First, we examined the associations with stratification of age group; then, demographic and socioeconomic variables (ethnicity, TDI, highest education level, and assessment center) were adjusted; furthermore, we additionally adjusted for lifestyle variables, including BMI, smoking status, alcohol consumption status, and physical activity. Finally, the associations were determined using a full adjusted model, including all covariates above and prevalent diagnoses of CVD, CKD, and COPD, and cancer survivorship duration. The associations between MetS with cause-specific mortality were examined using the competing risk model via the Fine-and-Gray method, accounting for other causes of death as a competing risk. The associations were quantified by estimating the hazard ratios (HRs) and the 95% confidence intervals (CIs). We further examined the association between the number of MetS components and the risk of mortality and incident comorbidities. Kaplan–Meier curve was plotted to depict the survival between survivors with and without MetS.

Subgroup analysis was performed for all outcomes of stratified age groups (<60 and ≥60 years), ethnicity (White and non-White), TDI (tiers 1, 2, and 3), highest education level (college and others), BMI (underweight or normal, overweight, and obesity), smoking status (never and previous or current), alcohol status (current and previous or never), and physical activity (low, moderate, high, and missing). The HRs and 95% CIs were estimated by fitting the interaction term of MetS-by-subgroup and the covariates into a Cox proportional regression model.

To assess the mediating biomarkers underlying MetS–mortality association, we first tested the association between NMR biomarkers and MetS. The absolute measures of biomarkers were log2-transformed and standardized using *z*-score. Multivariate logistics regression was employed to determine the association, adjusting for covariates mentioned above. The false discovery rate (FDR) was estimated and an FDR < 0.05 was claimed as significance. Then, we performed a mediation analysis considering NMR markers as potential mediators, using the same full adjustment model as the primary analysis. We estimated the average causal mediation effect (ACME), which represents the indirect effect through the NMR marker; the average direct effect (ADE), which represents the direct effect not through the NMR marker; the total effect, which represents the overall association between MetS and the outcome; and the proportion mediated, the ratio of the ACME to the total effect, which represents the proportion of the total effect explained by the NMR marker. Confidence intervals for mediation estimates were derived using non-parametric bootstrap resampling. *p-*values were adjusted using the Benjamini–Hochberg FDR method. Statistical significance for mediation was claimed when FDR < 0.05 for ACME and total effect.

All statistical analyses were performed using R software (version 4.3.1). Statistical significance was claimed when a two-tailed *p* < 0.05 unless otherwise specified.

## Results

### Characteristics of breast cancer survivors

The baseline characteristics of BC survivors are summarized in **Table 1**. A total of 6,027 BC survivors were included in this study, of whom the median age was 61 (IQR, 56 to 65) years old, the majority were White (*n* = 5,843, 96.9%), the median survivorship duration was 6.9 (IQR, 3.6 to 10.8) years, and 1,997 (33.1%) had MetS. Survivors with MetS had a lower socioeconomic status, including higher TDI [−1.20 (SD, 3.04) vs. −1.62 (2.84), *p* < 0.001] and lower education level [college, 430 (21.5%) vs. 1303 (32.3%), *p* < 0.001]. Unhealthy lifestyle was observed among survivors with MetS, with higher smoking rate [988 (49.5%) vs. 1697 (42.1%), *p* < 0.001], less physical activity [low, 365 (18.3%) vs. 499 (12.4%), *p* < 0.001], and poorer sleep [823 (41.2%) vs. 1,410 (35.0%), *p* < 0.001].

**Table 1:** Characteristics of breast cancer survivors.

Characteristics	Without MetS (*N* = 4,030)	With MetS (*N* = 1,997)	*p-*value
Age, years, median (IQR)	60 (55 to 64.0)	62 (58 to 66)	<0.001
Ethnicity, *n* (%)			0.44
White	3,915 (97.1%)	1928 (96.5%)	
Non-White	104 (2.58%)	62 (3.10%)	
TDI, mean (SD)	−1.62 (2.84)	−1.20 (3.04)	<0.001
Education, *n* (%)			<0.001
College	1,303 (32.3%)	430 (21.5%)	
Others	2,696 (66.9%)	1,536 (76.9%)	
BMI, kg/m^2^, median (IQR)	25.0 (22.9 to 27.4)	29.8 (27.2 to 33.1)	<0.001
Smoking status, *n* (%)			<0.001
Current or previous	2,315 (57.4%)	995 (49.8%)	
Never	1,697 (42.1%)	988 (49.5%)	
Alcohol status, *n* (%)			<0.001
Previous or never	321 (7.97%)	247 (12.4%)	
Current	3,701 (91.8%)	1,743 (87.3%)	
Physical activity, *n* (%)			<0.001
Low	499 (12.4%)	365 (18.3%)	
Moderate	1,363 (33.8%)	558 (27.9%)	
High	1,228 (30.5%)	425 (21.3%)	
Cancer survivorship, years, median (IQR)	7.0 (3.7 to 10.8)	6.8 (3.5 to 10.8)	0.522
Prevalent diagnosis, *n* (%)			
CVD	967 (24.0%)	1,030 (51.6%)	<0.001
AF	35 (0.87%)	28 (1.40%)	0.08
MI	20 (0.50%)	36 (1.80%)	<0.001
HF	8 (0.20%)	19 (0.95%)	<0.001
Stroke	44 (1.09%)	43 (2.15%)	0.002
COPD	86 (2.13%)	60 (3.00%)	0.048
CKD	38 (0.94%)	49 (2.45%)	<0.001
Depression	417 (10.3%)	253 (12.7%)	0.008

Missing: ethnicity [*n* = 18 (0.3%)], TDI [*n* = 5 (0.1%)], education [*n* = 62 (1.0%)], BMI [*n* = 10 (0.2%)], smoking status [*n* = 32 (0.5%)], alcohol status [*n* = 15 (0.3%)], and physical activity [*n* = 1,589 (26.4%)].

MetS, metabolic syndrome; IQR, interquartile range; TDI, Townsend Deprivation Index; SD, standard deviation; CVD, cardiovascular disease; AF, atrial fibrillation; MI, myocardial infarction; HF, heart failure; COPD, chronic obstructive pulmonary disease; CKD, chronic kidney disease.

### Association between MetS and mortality

**Figure 1:**
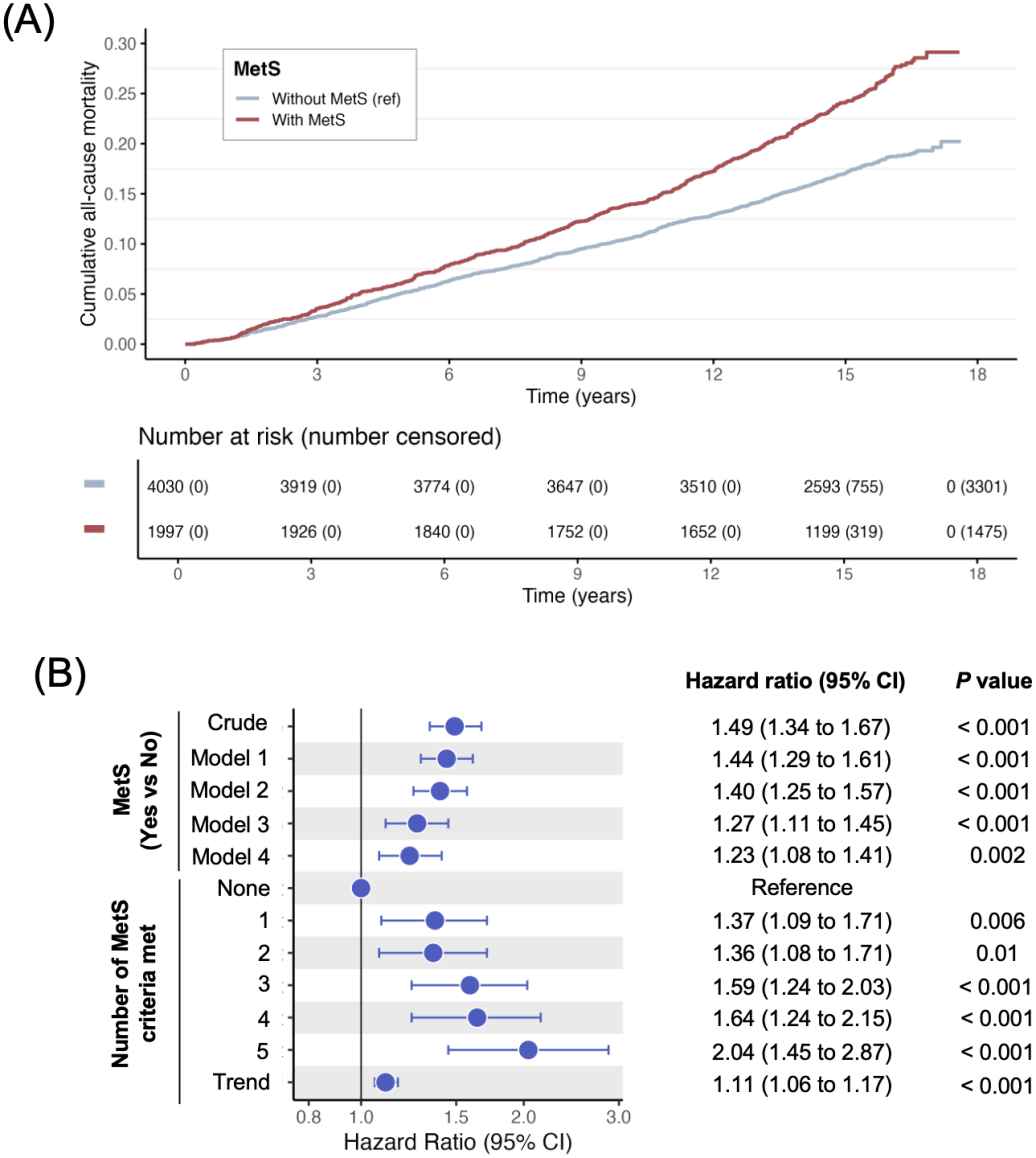
Association between MetS and all-cause mortality among breast cancer survivors. **(A)** Kaplan–Meier curve, **(B)** Forest plot demonstrating hazard ratios in different analyses. CI, confidence interval.

After a median follow-up of 15.7 (IQR 15.1 to 16.4) years, a total of 1,251 deaths were observed, of which 917 (73.3%) were cancer-specific and 101 (8.1%) were CVD-specific (**Supplementary Table 1**). MetS was significantly associated with elevated mortality risk among BC survivors (HR, 1.23; 95% CI, 1.08 to 1.41, *p* = 0.002) (**Figure 1A**). The greater number of MetS components survivors met, the higher risk of mortality was observed (**Figure 1B**). Subgroup analyses revealed that more pronounced risk was observed among BC survivors with overweight BMI (HR, 1.47; 95% CI, 1.22 to 1.77) and never-smoker survivors (HR, 1.40; 95% CI, 1.18 to 1.67) compared with their counterparts (*p* for interaction < 0.05) (**Supplementary Table 2**). Significant association was also observed for non-cancer-specific death (HR, 1.45; 95% CI, 1.13 to 1.86), but not for cancer-specific death (HR, 1.14; 95% CI, 0.97 to 1.34) or CVD-specific death (HR 1.27; 95% CI, 0.81 to 2.00) (**Table 2**). Sensitivity analysis adjusting lipid-, glucose-, and BP-lowering treatment demonstrated the robustness of the finding (HR, 1.21; 95% CI, 1.05 to 1.39, *p* = 0.007).

**Table 2:** Association between MetS with cause-specific mortality.

Models	Hazard ratio (95% CI)	*p*-value
Cancer-specific death		
Crude	1.26 (1.10 to 1.44)	0.001
Model 1	1.25 (1.09 to 1.43)	0.001
Model 2	1.23 (1.08 to 1.41)	0.003
Model 3	1.13 (0.96 to 1.33)	0.13
Model 4	1.14 (0.97 to 1.34)	0.10
MetS criteria count		
0	Reference	
1	1.21 (0.95 to 1.54)	0.13
2	1.25 (0.97 to 1.60)	0.08
3	1.39 (1.06 to 1.82)	0.02
4	1.33 (0.98 to 1.80)	0.07
5	1.52 (1.01 to 2.29)	0.04
Non-cancer-specific death		
Crude	2.20 (1.78 to 2.73)	<0.001
Model 1	1.97 (1.59 to 2.45)	<0.001
Model 2	1.84 (1.48 to 2.29)	<0.001
Model 3	1.65 (1.30 to 2.10)	<0.001
Model 4	1.45 (1.13 to 1.86)	0.003
MetS criteria count		
0	Reference	
1	2.68 (1.38 to 5.20)	0.004
2	2.39 (1.22 to 4.70)	0.01
3	3.20 (1.62 to 6.33)	0.001
4	3.65 (1.79 to 7.44)	<0.001
5	4.78 (2.21 to 10.31)	<0.001
CVD-specific death		
Crude	1.91 (1.29 to 2.81)	0.001
Model 1	1.71 (1.15 to 2.55)	0.01
Model 2	1.65 (1.11 to 2.44)	0.01
Model 3	1.51 (0.98 to 2.34)	0.06
Model 4	1.27 (0.81 to 2.00)	0.30
MetS criteria count		
0	Reference	.
1	2.63 (0.78 to 8.92)	0.12
2	2.89 (0.86 to 9.78)	0.09
3	3.19 (0.90 to 11.28)	0.07
4	3.36 (0.90 to 12.60)	0.07
5	4.85 (1.21 to 19.49)	0.03

CI, confidence interval.

Model 1 was stratified by age group; model 2 was additionally adjusted for sociodemographic variables (ethnicity, TDI, highest education level, and assessment center); model 3 was additionally adjusted for lifestyle variables (BMI, smoking status, alcohol consumption status, and physical activity); model 4 was additionally adjusted for health status (prevalent diagnoses of CVD, CKD, and COPD, and cancer survivorship duration).

### Association between MetS and comorbidity incident

The association between MetS and comorbidity incident is summarized in **Table 3**. Significant association was observed for MetS with MI (HR, 1.56; 95% CI, 1.02 to 2.4) and AF (HR, 1.61; 95% CI, 1.04 to 2.49), but not for other CVDs [HR (95% CI), stroke, 1.25 (0.88 to 1.78), and HF, 1.23 (0.94 to 1.62)]. Similarly, the greater number of MetS components survivors met, the higher risk of MI and AF was observed. A remarkable elevated risk was also observed for BC survivors with MetS for CKD incident (HR, 1.98; 95% CI, 1.56 to 2.51), with almost 4.5 times increased risk among survivors who met all MetS criteria (HR, 4.5; 95% CI, 2.22 to 9.13). No significant association was observed for COPD (HR, 1.23; 95% CI, 0.93 to 1.61) or depression (HR, 1.09; 95% CI, 0.84 to 1.42) incident. Subgroup analyses regarding comorbidity incident were demonstrated in **Supplementary Table 2**. Survivors with overweight BMI were at higher risk of HF incident (HR, 2.22; 95% CI, 1.32 to 3.73, *p* for interaction = 0.02), while those with current or previous smoking status were more prone to incident COPD (HR, 1.22; 95% CI, 0.92 to 1.63, *p* for interaction < 0.001).

**Table 3:** Association between MetS with comorbidity incident.

Models	Hazard ratio (95% CI)	*p*-value
Stroke		
Full adjusted model	1.25 (0.88 to 1.78)	0.21
MetS criteria count		
0	Reference	
1	1.56 (0.82 to 2.96)	0.18
2	1.44 (0.74 to 2.79)	0.28
3	1.81 (0.91 to 3.61)	0.09
4	1.57 (0.72 to 3.40)	0.26
5	2.62 (1.07 to 6.41)	0.03
Atrial fibrillation		
Full adjusted model	1.61 (1.04 to 2.49)	0.03
MetS criteria count		
0	Reference	
1	1.31 (0.56 to 3.09)	0.54
2	1.39 (0.59 to 3.31)	0.45
3	1.69 (0.69 to 4.13)	0.25
4	2.61 (1.03 to 6.62)	0.04
5	3.98 (1.4 to 11.31)	0.01
Myocardial infarction		
Full adjusted model	1.56 (1.02 to 2.4)	0.042
MetS criteria count		
0	Reference	.
1	1.30 (0.55 to 3.06)	0.55
2	1.49 (0.63 to 3.51)	0.36
3	1.72 (0.71 to 4.18)	0.23
4	2.60 (1.03 to 6.58)	0.04
5	3.88 (1.37 to 11.00)	0.01
Heart failure		
Full adjusted model	1.23 (0.94 to 1.62)	0.137
MetS criteria count		
0	Reference	
1	0.91 (0.54 to 1.51)	0.71
2	0.95 (0.57 to 1.58)	0.85
3	1.17 (0.70 to 1.98)	0.55
4	0.93 (0.52 to 1.67)	0.82
5	1.90 (1.00 to 3.58)	0.048
COPD		
Full adjusted model	1.23 (0.93 to 1.61)	0.144
MetS criteria count		
0	Reference	
1	1.32 (0.77 to 2.28)	0.31
2	1.80 (1.05 to 3.09)	0.03
3	1.70 (0.96 to 3.02)	0.07
4	2.30 (1.26 to 4.21)	0.01
5	2.07 (0.98 to 4.34)	0.06
CKD		
Full adjusted model	1.98 (1.56 to 2.51)	<0.001
MetS criteria count		
0	Reference	.
1	1.58 (0.84 to 2.96)	0.15
2	2.01 (1.08 to 3.74)	0.03
3	2.99 (1.60 to 5.58)	0.001
4	4.29 (2.26 to 8.15)	<0.001
5	4.50 (2.22 to 9.13)	<0.001
Depression		
Full adjusted model	1.09 (0.84 to 1.42)	0.52
MetS criteria count		
0	Reference	.
1	1.19 (0.78 to 1.81)	0.43
2	1.14 (0.73 to 1.77)	0.56
3	1.17 (0.73 to 1.89)	0.51
4	1.39 (0.83 to 2.35)	0.22
5	1.27 (0.64 to 2.51)	0.50

CI, confidence interval.

Full adjusted model was stratified by age group, and adjusted for sociodemographic variables (ethnicity, TDI, highest education level, and assessment center), lifestyle variables (BMI, smoking status, alcohol consumption status, and physical activity), and health status (prevalent diagnoses of CVD, CKD, and COPD, and cancer survivorship duration).

### Mediation analysis of metabolomes

**Figure 2:**
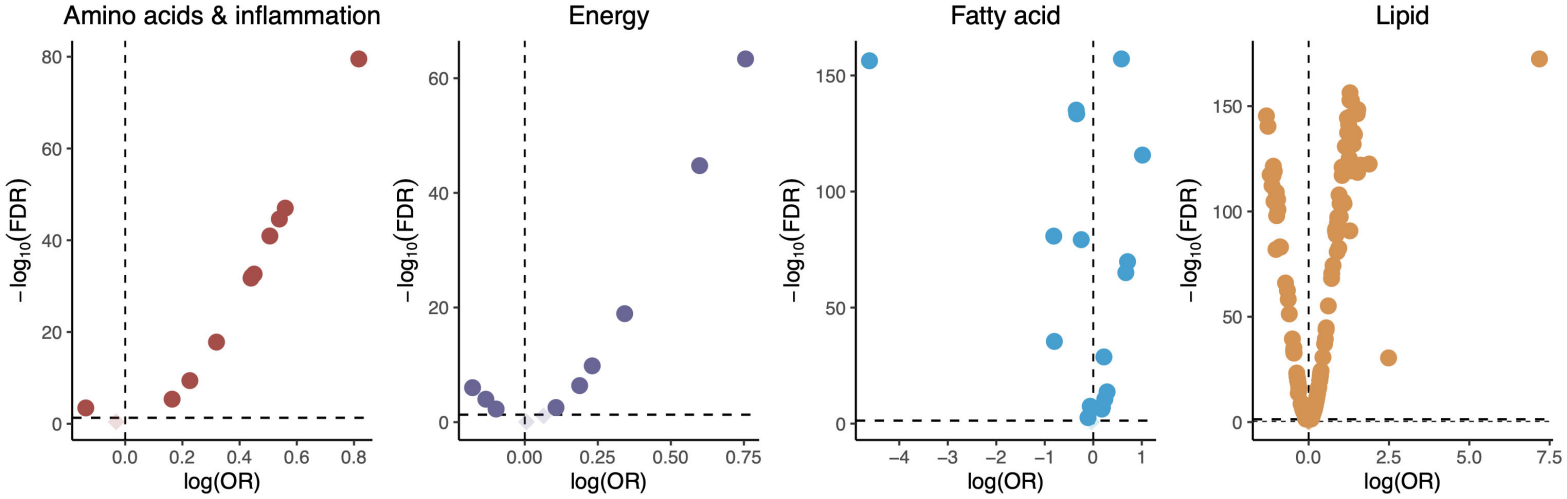
Association between MetS and metabolic biomarkers among breast cancer survivors. A false discovery rate (FDR) of less than 0.05 was considered as statistical significance. FDR, false discovery rate; OR, odds ratio.

The association between MetS and NMR biomarkers is summarized in **Supplementary Table 3**; **Figure 2**. Among survivors with MetS, inflammation status [glycoprotein acetyls, OR (95% CI), 2.26 (2.08 to 2.46)] and poorer renal function [creatinine, 1.41 (1.31 to 1.52)] were observed. Also, fatty acid (FA) profiles showed a consistent pattern of dysregulation in relation to risk. Higher levels of total FAs, monounsaturated FAs (MUFA), and saturated FAs (SFA) were associated with MetS, with the strongest association observed for MUFA (OR, 2.76; 95% CI, 2.53 to 3.01). In contrast, markers reflecting greater FA unsaturation demonstrated inverse associations. Lower ratios of polyunsaturated FAs (PUFA) to total FAs (OR, 0.71; 95% CI, 0.69 to 0.73) and docosahexaenoic acid (DHA) to total FAs (OR, 0.45; 95% CI, 0.40 to 0.51) were associated with MetS. Notably, the ratio of PUFA to MUFA exhibited the most pronounced inverse association (OR, 0.01; 95% CI, 0.007 to 0.014). Also, survivors with MetS demonstrated high VLDL measurements and low HDL measurements.

Mediation analysis identified FA composition and inflammation as the potential mediating factors between MetS and mortality (**Supplementary Table 4**). The ratio of PUFA to total FAs (63.0%) and the PUFA-to-MUFA ratio (58.2%) showed the largest mediation proportions, followed by triglycerides-to-total lipids ratio in small HDL (55.6%) and GlycA (51.7%) (all FDR < 0.05 for both ACME and total effect).

## Discussion

Leveraging data from UK Biobank, a large-scale, prospective cohort study, we found that MetS was associated with significantly elevated all-cause mortality and non-cancer-specific mortality among BC survivors. Also, increased risks in MI, AF, and CKD incident were also observed for survivors with MetS. Subgroup analyses suggested that survivors with lower socioeconomic status and unhealthy lifestyle were at higher risk. Mediation analyses revealed that PUFA and inflammation were the potential mediating factors between MetS and mortality.

MetS has been reported to be associated with a range of adverse health outcome among the general population (4–8). Here, to the best of our knowledge, our study was the largest one focused on BC survivors, examining both mortality and comorbidity incidents, revealing a potential underlying metabolic pathway. Consistent with our findings, MetS was reported to be associated with elevated mortality in non-European BC survivors (9). Of note, we found that the excess mortality risk was primarily attributed to non-cancer-specific death. This finding suggested a critical but often underrecognized aspect of survivorship, of which long-term prognosis in BC survivors was increasingly shaped by competing non-cancer risks rather than cancer recurrence alone. It highlighted the importance of metabolic health as a key determinant of survival, suggesting that MetS may serve as a central, modifiable driver of adverse outcomes other than oncologic outcomes. These results support the integration of metabolic risk stratification and management into routine survivorship care, including dietary intervention and physical activity (18–20). Of note, our findings have substantial clinical relevance among BC survivors. Approximately 70% of BC cases are hormone receptor-positive, for which most patients require long-term endocrine therapy (21). This prolonged endocrine treatment course may exacerbate metabolic disturbances, including dyslipidemia, insulin resistance, and weight gain (11, 12). MetS was more common among this vulnerable population compared with others.

Also, we expanded the evaluation of MetS beyond mortality to include comorbidity incident, given the substantial and clinically meaningful disease burden accompanying prolonged survivorship driven by advances in cancer treatment. Compared with another study (22), an increased risk in overall CVD incident was not observed, potentially due to the different definition of study outcome. Yet, increased risk in MI incident and AF incident was found. This discrepancy suggests a heterogeneous impact of MetS across cardiovascular outcomes, where composite endpoints may obscure associations with specific disease phenotypes. The observed associations with MI and AF indicate that MetS might preferentially promote atherosclerosis and cardiac remodeling. These effects may be further amplified in BC survivors due to metabolic disturbances. Meanwhile, elevated CKD risk was also observed. Our findings highlighted the importance of examining specific cardiovascular and renal outcomes and underscored the need for targeted cardiometabolic management in this population.

Furthermore, our mediation analysis identified that PUFA-related measures and systemic inflammation play key mediating roles linking exposure to adverse outcomes. Reduced PUFA levels, particularly those reflecting lower FA unsaturation, may indicate a shift toward a more pro-inflammatory lipid profile, while elevated inflammatory markers such as GlycA reflect inflammation (23, 24). Together, these pathways suggest that disrupted lipid metabolism and persistent inflammation may act synergistically to drive the increased risk observed, providing biologically plausible targets for intervention (25, 26).

Our study has several limitations. First, the majority population of this study was White, who were more socially advantaged to health resources than the general population, which may limit the generalizability of the conclusions. Second, although we adjusted for a diverse of covariates, several confounding, such as diet and other pre-existing disease background, could not be fully captured. Therefore, findings in our study should be interpreted with caution. Third, as this was an observational study, the findings in the mediation analysis could not be interrupted as evidence of a causal effect or causal pathway. Although our results suggest a potential mediating role, replication and validation in independent cohorts and studies with more robust causal inference designs are needed. Therefore, the observed mediation should be interpreted with caution. Fourth, consistent with other studies (16, 27), although BC survivors were defined as individuals diagnosed at least 1 year before enrollment, some participants may still have been receiving active treatment at baseline. Because of incomplete treatment-phase and molecular subtype data and the population-based design of UK Biobank, tumor characteristics and active treatment status could not be fully determined. Despite adjustment for survivorship duration, potential selection bias and residual confounding may remain. Further studies capturing treatment information and focusing on BC survivors are warranted. Fifth, the fact that 26.4% of survivors had missing information on physical activity makes the imputation unreliable. Physical activity was adjusted for as a categorical covariate, with missing values included as a separate category, which may still have introduced residual confounding. Finally, the ascertainment of MetS was based on baseline assessment. The dynamic in metabolic status caused by aging, treatment, and health behavior change could still affect the findings, although we have adjusted for these covariates at baseline. Further longitudinal studies are needed to assess the effect of dynamic change in metabolic status on health outcomes.

## Conclusion

Utilizing data from the UK Biobank cohort, we found that MetS was significantly associated with elevated mortality and increased incident of MI, HF, and CKD among BC survivors, which were more pronounced among survivors with lower socioeconomic status and unhealthy lifestyle. Disrupted FA and systemic inflammation were identified as the potential mediators underlying the association between MetS and mortality. Findings highlighted that MetS as a potentially modifiable risk factor in BC survivors, underscoring the need for targeted metabolic management to improve long-term outcomes.

## Data Availability

The original contributions presented in the study are included in the article/**Supplementary Material**. Further inquiries can be directed to the corresponding author/s.
